# Distributive Shock in a Case of Erythrodermic Psoriasis Associated With Bacteremia From Multiple Opportunistic Pathogens: A Case Report

**DOI:** 10.7759/cureus.99131

**Published:** 2025-12-13

**Authors:** Halit Özel

**Affiliations:** 1 Intensive Care Unit, Université Libre de Bruxelles, Brussels, BEL

**Keywords:** bacterial septicemia, distributive shock, erythrodermic psoriasis, intensive care unit, opportunistic bacterial infection

## Abstract

Erythrodermic psoriasis is a specific form of psoriasis that can present with life-threatening complications and complex management. Circulatory shock and bacterial infection related to the skin lesions are among the causes that can lead to the patient's death. Furthermore, alterations of these patients’ bacterial flora could be involved in the development of the pathology and its complications. We report here the case of a 59-year-old female with erythrodermic psoriasis who presented with severe distributive shock and bacteremia caused by multiple opportunistic pathogens, resulting in a fatal outcome in less than 48 hours.

## Introduction

Psoriasis is an inflammatory skin disease with an incidence ranging from 0.5% to 11.4% worldwide [1]. The pathophysiological mechanism results from a complex interaction between the skin and environmental, genetic, and autoimmune factors [2]. Erythrodermic psoriasis (EP), which manifests as erythema covering at least 75% of the body surface area, is an unusual and potentially fatal form of the disease, particularly due to impaired skin barrier function leading to infection and septic shock, protein and fluid loss, and thermoregulatory disruption [3]. It represents less than 3% of psoriasis cases, and mortality remains high [3,4].

Alteration of the skin microbial flora with a change in the representation of the different bacteria, particularly *Corynebacterium simulans*, is reported in the literature [5], although there is still no formal proof between the pathophysiology of psoriasis plaques and this disturbance of the flora [6]. The association of EP with septicemia, particularly staphylococcal and opportunistic pathogens, is a rare and poorly documented phenomenon [7,8]. We report here the case of a patient presenting to the hospital with severe circulatory shock caused by EP complicated by opportunistic pathogen septicemia.

## Case presentation

A 59-year-old female patient, living alone at home, with a known history of psoriasis (without follow-up) and high blood pressure, presents to the emergency department following a deterioration in her general condition associated with a skin rash for 48 hours. Initial findings revealed arterial hypotension with unrecordable blood pressure, associated with severe hypothermia (27°C). The patient was also tachypneic and obtunded, preventing a complete and reliable medical history. Her skin was marked by an exfoliative erythematous rash affecting 80% to 90% of her body.

Laboratory findings are summarized in Table 1. Initial blood tests revealed a C-reactive protein (CRP) of 390 mg/L and acute kidney injury with a glomerular filtration rate (GFR) of 7 mL/min/1.73 m². Other blood findings were inconclusive, including autoimmune panel, viral and bacterial serologies, thyroid function tests, and cortisol levels. Venous blood gas analysis on admission showed an initial lactate level of 5.6 mmol/L and marked metabolic acidosis with a pH of 7.19. Extensive bacterial cultures were performed. Echocardiography on admission showed normal systolic and diastolic function. Standard chest X-ray imaging was unremarkable.

**Table 1:** Initial Laboratory Findings TSH: thyroid-stimulating hormone.

Parameter	Result	Reference Range
Hemoglobin	12.3 g/dL	11.7-16.0 g/dL
White blood cells	8,300/mm³	4,500-11,000/mm³
C-reactive protein	390 mg/L	<5 mg/L
Sodium	138 mmol/L	136-145 mmol/L
Potassium	4.8 mmol/L	3.5-5.1 mmol/L
Urea	339 mg/dL	21-43 mg/dL
Creatinine	7.7 mg/dL	0.50-0.90 mg/dL
Glomerular filtration rate	7 ml/min/1.73 m²	>60 ml/min/1.73 m²
TSH	0.67 mU/L	0.27-4.20 mU/L
T4	12.7 pmol/L	12.0-22.0 pmol/L
Cortisol AM (6-10 AM)	529 nmol/L	133-537 nmol/L
Venous blood pH	7.19	7.32-7.43
Lactate	5.6 mmol/L	0.56-1.39 mmol/L

Initial management at the emergency department started with fluid resuscitation using crystalloid solution and empirical antibiotic therapy with ceftriaxone and clindamycin, along with active warming efforts. As the patient's hemodynamic status remained unstable with unrecordable blood pressure, she was transferred to the intensive care unit (ICU) where vasopressor support with norepinephrine and hydrocortisone was initiated. Given the renal insufficiency with metabolic acidosis and anuria, dialysis was also started within the first hours of admission in the ICU. The empirical antibiotic regimen was adjusted within the same timeframe by adding vancomycin.

A few hours later, the patient's condition deteriorated rapidly, requiring sedation and mechanical ventilation, as well as the addition of a second vasopressor, glypressin, and inotropic support with dobutamine. The antimicrobial treatment remained unchanged, but we decided to empirically treat possible fungemia with intravenous diflucan. A skin sample was taken using a punch biopsy on the same day. No specific treatment for psoriasis was proposed, in consultation with dermatologists, considering the severity of the predominant and uncontrolled shock.

**Figure 1:**
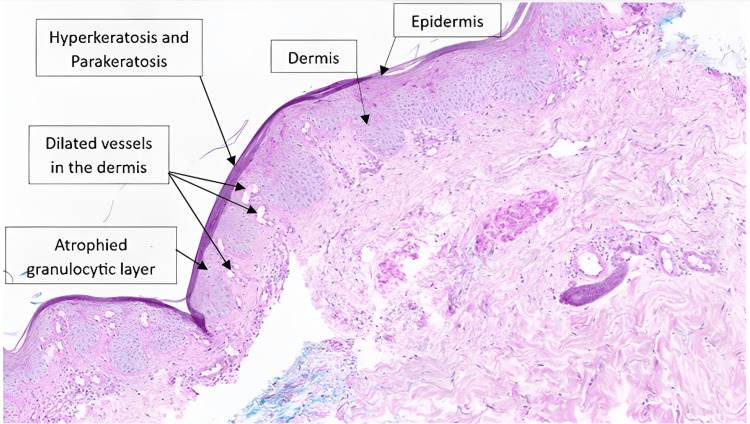
Histological Analysis 10x Zoom, Hematoxylin-Eosin Staining The biopsy showed an epidermis surmounted by hyperplasic, compact and parakeratotic stratum corneum, with an atrophied granulocytic layer. The basal layer is infiltrated by sparse lymphocytic elements. The papillary dermis contains dilated vessels and a mild to moderate lymphomonocytic inflammatory infiltrate. Hyperkeratosis (thickening of the stratum corneum) and parakeratosis (presence of nuclei within corneocytes) are very common in psoriasis. Atrophy of the granulocytic layer is also a sign of psoriasis and is linked to abnormal keratocyte differentiation. Dilated blood vessels and lymphocytic infiltration in the papillary dermis are typical inflammatory changes in psoriasis.

On the second day, the patient's condition remained unfavorable, with multiple organ failure and no response to vasopressors and inotropic therapy. She died the same day despite all resuscitation efforts. Subsequent microbiological tests revealed positive blood cultures for *Corynebacterium simulans*, *Pseudomonas putida*, and *Finegoldia magna*. Furthermore, we also detected *Corynebacterium simulans* on skin smears. Histological analysis of the skin biopsy revealed an epidermis covered by a hyperplasic and parakeratotic stratum corneum, alongside with other findings, consistent with psoriasis (Figure 1).

## Discussion

EP is a rare form of psoriasis with a potentially fatal outcome, particularly due to impaired skin barrier function [3,4]. Associated complications are numerous, including hemodynamic, metabolic, respiratory, and infectious complications [7,9,10]. We have reported here the case of a patient with complicated EP in the foreground of distributive shock associated with septicemia from several opportunistic germs, the outcome of which was fatal.

Green et al. [7] reported a series of five cases of EP complicated by septicemia, each time due to *Staphylococcus aureus*. Interestingly, two of these cases involved patients with human immunodeficiency virus (HIV), which is likely a significant factor. They reported two deaths, one of which was among the HIV-positive patients. This contrasts with our patient, who presented with septicemia caused by multiple opportunistic pathogens. Richardson et al. [8], on the other hand, reported a case of EP complicated by bacteremia and fungemia, thus highlighting the possibility of involvement by opportunistic pathogens.

*Corynebacterium simulans​* is a commensal Gram-positive bacillus of the skin first isolated in 2000 by Wattiau et al. [11]. It is a bacterium found in abundance in the skin flora of patients with psoriasis, and the role of the interaction between the immune system and these germs in the development of the pathology remains uncertain [12]. The *Finegoldia magna*, formerly known as *Peptostreptococcus* *magnus*, is a Gram-positive anaerobic cocci that colonizes the skin and mucous membranes. It is an opportunistic pathogen as reported by Murdoch et al. [13]. *Pseudomonas putida* belongs to the group of fluorescent pseudomonas, a group of opportunistic pathogens associated with nosocomial infections [14]. It is among the suspected pathogens that can cause an exacerbation of psoriatic eruptions [15]. In the largest series to date, reported by Yang et al. [16], only two of 55 cases were associated with a skin infection; eight cases of sepsis were also noted.

To date, no cases of septicemia caused by these bacteria have been reported in patients with EP. Therefore, the present case, by demonstrating septicemia caused by these different bacteria, reinforces previously put forward hypotheses concerning the involvement of microbiota changes in the pathogenicity of psoriasis. EP is associated with a mortality rate ranging from 9% to 64% [3,17]. In the series of septicemia reported by Green et al. [7], two of the five cases resulted in death. The cause of death was not mentioned. In our patient's case, hemodynamic instability with a lack of response to vasopressors and inotropes led to her death.

It can be assumed that distributive shock associated with EP, with or without a septic component, is the primary cause of death. Only a few isolated cases of distributive shock in patients with EP have been reported in the literature [8,18]. As can already be seen in the previous cases, it is difficult to clearly distinguish the septic component from the component purely related to psoriasis in terms of its contribution to the severity of distributive shock.

We found no clear consensus in the literature for the treatment of distributive shock in the context of EP; therefore, the current treatment is no different from that applied in cases of distributive shock. In our case, we had to rapidly administer support with norepinephrine, glypressin, and dobutamine, depending on the evolution of the severity of hemodynamic instability, as recommended in the literature for shocks [19]. The choice of empirical antibiotic therapy was made to ensure broad coverage, including methicillin-resistant *Staphylococcus aureus*, by combining ceftriaxone, clindamycin, and vancomycin. Given the severity of the condition, we opted to add diflucan to cover potential fungemia. No treatment for psoriasis was proposed for our patient, in consultation with the dermatologists. Indeed, there is no clear recommendation in the literature regarding severe EP with shock. The patient died before receiving the results of the blood cultures, which ultimately revealed a series of opportunistic germs, which is consistent with the changes in bacterial flora and immune alteration demonstrated in patients with psoriasis.

## Conclusions

EP is a rare and potentially fatal form of psoriasis, the management of which can be complex. Several complications can occur, including bacterial superinfections and shock, which can be septic or purely related to EP, with the distinction between the two being particularly challenging. Changes in the bacterial flora associated with psoriasis and immune system alterations may contribute to these complications. It is important to be aware of these factors and consider the possibility of infection with opportunistic pathogens. The situation can deteriorate rapidly, so treatment must include broad-spectrum antibiotic therapy and follow rigorous resuscitation measures.
